# Ion chromatography coupled to Q-Orbitrap for the analysis of formic and oxalic acid in beehive matrices: a field study

**DOI:** 10.1007/s00216-022-03882-2

**Published:** 2022-02-17

**Authors:** Icíar Beraza Gómez, María José Gómez Ramos, Łukasz Rajski, José Manuel Flores, Florencia Jesús, Amadeo R. Fernández-Alba

**Affiliations:** 1 Department of Chemistry and Physics, University of Almería, Agrifood Campus of International Excellence ceiA3 (ceiA3), Carretera Sacramento s/n, La Cañada de San Urbano, 04120 Almería, Spain; 2 Department of Zoology, University of Córdoba, Campus of Rabanales, 14071 Córdoba, Spain; 3 Grupo de Análisis de Compuestos Traza, Polo de Desarrollo Universitario “Abordaje holístico”, CENUR Litoral Norte Sede Paysandú, Universidad de la República, Ruta 3 km 363, 60000 Paysandú, Uruguay

**Keywords:** Natural acaricides, Organic acids, Veterinary treatments, IC-HRMS, Polar compounds, Beekeeping samples

## Abstract

There is an increasing concern about the use of synthetic acaricides to fight the ectoparasitic mite *Varroa destructor*. Natural products such as formic acid (FA) and oxalic acid (OA) have emerged as a possible alternative control strategy. However, given the difficulty of analysing these highly polar compounds and the lack of robust and reliable methods, there are very few studies of the concentration and distribution of these natural acaricides in the beehive compartments. We present a reliable and simple analytical methodology, based on sample extraction with modified quick polar pesticide (QuPPe) methods followed by ion chromatography coupled to a quadrupole Orbitrap mass analyser for the analysis of FA and OA in honeybees, honey, beeswax, and beebread. The developed methods have been used in a field study for the evaluation of the presence and distribution of FA and OA in the beehive products, as well as in adult bees and bee brood samples, before, during, and up to 3 months after the application of the treatments by the beekeeper. Beebread and honey samples presented the highest concentration levels of OA and FA, respectively, mainly due to their natural presence. As expected, the organic acids showed low persistence in wax after the treatments. The natural acaricides were found in adult and developing bees at concentration levels below the reported LD50 in all the cases; however, residue levels of OA in larvae during the treatment application were very close to the reported LD50.

**Graphical abstract:**

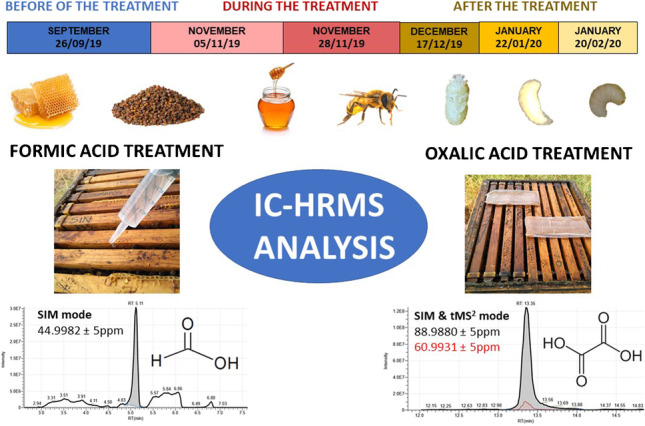

## Introduction

The western honeybee *Apis mellifera* ranks among the most important pollinators worldwide [1]. They provide key ecosystem services for global agriculture [2]. Beekeeping is an important source of income because of the honeybee role in collecting and manufacturing products such as honey and beeswax [3]. Over the last few decades, there seems to be a decrease in honeybee colonies in both Europe and the USA [1, 4]. In temperate regions, the invasive species of *Varroa* mite could be a major threat to honeybee populations. *Varroa destructor* produces several adverse effects such as a reduction of bee’s immune response [5]. In the recent past, the most common treatments applied to fight *V. destructor* are based on the use of chemicals: synthetic chemicals such as coumaphos, fluvalinate, and amitraz, which are relatively inexpensive and easy to apply [5]. Nonetheless, the efficacy of these compounds is limited because of the contamination of hive products as consequence of accumulation of pesticide residues [1, 6, 7], which could lead to the development of pesticide resistance in *V. destructor* populations reducing their efficacy [8]. It has been found that residues of synthetic acaricides could be a cause of colony losses by affecting the colony’s health and development [9]. Lethal and sub-lethal effects in honey bees may happen due to chronic exposure of repeated ingestion or contact with a certain chemical [10, 11]. For these reasons, an alternative to chemical treatments is needed to fight *Varroa* mites and ensure the honey

bees’ health.

Recently, beekeeping industry has focused on the use of naturally occurring chemicals for the control of *V. destructor* in attempt to find more “natural” and non-toxic substances [12, 13]. Essential oils of several plants are used to control *Varroa* mites in honeybee colonies; among them, Thyme (*Thymus vulgaris*) oil, which is rich in thymol and carvacrol [13–16]. However, residues of thymol may be accumulated in bee products (beeswax, honey, and bee pollen) after its application [13, 17]. Although they do not seem to be toxic for humans, their possible effects on the quality of the products could be a reason of important concern; it has been demonstrated a change in the taste of honey due to the presence of thymol [16, 18]. Organic acids such as FA (formic acid) and OA (oxalic acid) are the naturally occurring chemicals most used by beekeepers to fight the mite *V. destructor*. These organic acids are allowed for use in natural beekeeping and according to EU residue regulation for foodstuffs of animal origin, the establishment of a MRL (maximum residue level) for OA and FA is not required for all food produced by bees [12, 19]. These compounds have a high efficacy against *V. destructor* [3, 20, 21]*.* Moreover, there is no evidence that mite populations had developed resistance to organic acaricides [3, 5, 22].

No field experiments have been carried out measuring the probable residues after treatment with FA and OA in beekeeping, which makes it necessary to assess these effects on the quality of bee products [23]. While accumulation in wax is unlikely due to the hydrophilic character of organic acids, residual contamination may occur in honey [5, 24]. Furthermore, these organic acaricides have relatively low toxicity to bees, but excessive use by beekeepers can potentially lead to the weakening of the bee colony [25]. There are only a few studies dealing with the exposure of bee brood to these organic acids. It has been reported that application of OA and FA could increase cell death in bee larvae [26, 27].

The analysis of these organic acids is a very difficult task, as they are highly hydrophilic with low molecular weight. To date, it has been reported a variety of methods for OA separation and detection, including spectrophotometry, hydrophilic interaction liquid chromatographic (HILIC), ion chromatography, chemiluminescence, and enzymatic methods based on oxalate oxidase and oxalate decarboxylase [28]. As a powerful analytical method, ion chromatography (IC) can separate small organic acids such as FA in food and beverage samples [21, 29]. Different detectors are used with IC, such as UV, conductivity, and mass detectors [21, 30]. Recently, IC coupled to a quadrupole Orbitrap mass analyser has been successfully applied for the analysis of highly polar pesticides and their metabolites in vegetable matrices and honey [30, 31]. However, even though FA and OA are widely used in beekeeping, there are very few methods for the determination of these organic acaricides in the different beehive matrices. Most of the studies are focused on honey or adult bees. Kasiotis et al. developed two analytical methodologies to determine OA in bee bodies; one of them was an HPLC-photo diode array (PDA) method using a zwitterionic (ZIC)-hydrophilic interaction liquid chromatographic (HILIC) column and the other one was a gas chromatography coupled to mass spectrometry (GC-MS) method after OA derivatization [25]. Other study determines OA in honey and bees by ion exclusion chromatography and UV-VIS diode array detector [32]. Ion chromatography with conductometric detection was used for the analysis of FA in bee honey [21].

Considering the above-mentioned characteristics, this work aims to develop and apply analytical methodologies to robustly analyse FA and OA in bees and beehive matrices by IC coupled to high-resolution MS using a quadrupole (Q)-Orbitrap instrument and simple and efficient extraction methods. The methodology is fast and straightforward. In addition, the purpose of this study is the evaluation of the distribution and dissipation of FA and OA residues in adults and developing bees, honey, beeswax, and beebread samples before, during, and after the administration of treatments in field conditions. To our knowledge, there are no reports that evaluate comprehensively, in field conditions, the presence and distribution of these organic acids in the beehive and their products after administration of the treatments.

## Experimental

### Reagents and materials

High-purity standard solutions of formic and oxalic acids (Purity > 99.5%) were purchased from Sigma-Aldrich (Germany) and were stored at −30 °C. 2,4-D^13^C6. (used as internal standard) was obtained from Lab standards (Budapest, Hungary).

Individual stock solutions (1000 mg/L) of formic and oxalic acids were prepared in water and methanol and were stored in plastic vials in the dark at −20 °C. A mixed-standards solution was prepared from the stock standards. Water was obtained from Fisher Scientific (Fair Lawn, NJ) and methanol from Fluka Analytical (Steinheim, Germany). The Pierce^TM^ FlexMix^TM ^Calibration Solution was provided by Thermo Fisher Scientific (Rockford, IL, USA).

A Sonoplus HD 3100 ultrasonic system supplied by Bandelin Electronic GmbH & Co. KG (Germany) was used. It was equipped with a GM 3100 high-intensity generator (100 W), a UW 3100 ultrasonic converter, an SH 70G standard horn, and a 3-mm-diameter titanium MS73 probe for 2–50 mL volumes. An AGYTAX® automatic axial extractor supplied by Cirta Lab. S.L. (Spain) was also employed.

### Field study and sampling

The experiment was made from 12 honeybee colonies of a research station apiary located at the University of Córdoba (37° 55′33.5″N, 4° 43′26.1″W), South of Spain. More details about the apiary location can be found elsewhere [11]. For this study, conventional commercial wax was used. The beehives were placed on platforms raised 50 cm above ground level and maintained by the beekeeper.

The study consisted of 4 colonies treated with OA, 4 colonies treated with FA, and 4 untreated colonies used as control. The treatments were applied twice, in two consecutive months. OA treatment consisted in supplying a syrup (sugar solution) with the treatment poured with a syringe into the spaces between the beehive frames, to wet the bees (Ecoxal®, 1.65 gr of OA per beehive). In contrast, FA treatment (was applied with two sustained-release strips on top of the combs (Maqs®, 136.4 gr of FA per beehive). The study was carried out from September 2019 to February 2020. During the essay, the bee colonies were sampled six times. The first sampling (S1) was carried out in September, before the treatment application. The second sampling (S2) was carried out 3 weeks after the first treatment application, just before the application of the second dose treatments (November 5^th^, 2019). The third sampling (S3) was performed after the completion of the treatments (November 28^th^, 2019). The fourth (S4, December 17^th^, 2019), fifth (S5, January 22^nd^, 2020), and sixth sampling (S6, February 20^th^, 2020) were accomplished, 1, 2, and 3 months after the removal of the second treatment strips, respectively. Adult bees were sampled three times, before the treatment application, during the treatment, and 3 months after the treatment

Pieces of combs (approx. 25 cm^2^) containing beeswax, beebread, honey, bees, and bee brood (larvae, prepupae, and pupae) from each beehive were collected for the analysis. In the laboratory, the beebread, honey, and bee brood were carefully separated from the beeswax manually, in the case of honey using a filter. All the samples were labelled accordingly and quickly stored at −20 °C prior to the analyses. Samples from the 8 honeybee colonies treated and samples for the 4 colonies untreated (control) were pooled for their analysis.

### Sample preparation

#### Larvae, prepupae, pupae, and adult bees

Sample treatment was carried out using a modified Quick Polar Pesticide Method for products of Animal Origin (QuPPe-AO-Method) [33]. The extraction of analytes from the bodies of bees was facilitated using ultrasonic-assisted probe extraction, based on the method proposed by Gil et al. for the analysis of pesticides in honeybees [34]. First, 2 g portion of bee brood sample was weighed into a 50 mL PTFE centrifuge tube. Next, an appropriate volume of water was added until 10 mL (8.7 mL for bees, 8.6 mL for larvae, and 8.4 mL for prepupae) and samples were manually shaken. Then, samples were sonicated and shaken in an automatic axial extractor (AGYTAX®; Cirta Lab S.L., Madrid, Spain) for 10 min. The extract was centrifuged (3700 rpm) for 5 min. The following step was a dispersive solid-phase extraction (DSPE) clean-up step in which extracts were transferred to a 15 mL Falcon tube containing 100 mg of Bondesil-C_18_ and 2 mL of Acetonitrile and were vortexed for 1 min. Samples were then centrifuged for 5 min (3700 rpm). Finally, 1 mL of supernatant was transferred to a plastic vial (plastic vials are recommended as some compounds tend to interact with glass).

The concentration of OA and FA is given in two different units: mg/kg bee brood and ng per body. To obtain the concentration values in terms of ng per body, 2 grams of bee and bee brood samples of each kind were weighed (3 replicates) and the mean weight per larvae, prepupae, pupae, and adult bee body was calculated as 104, 126, 118, and 113 mg, respectively.

#### Honey

The extraction method for honey samples was a dilution with agitation at 35°C and shoot. A 5 g portion of homogenized sample was weighed into a 50 mL PTFE centrifuge tube. Next, an appropriate volume of ultrapure water was added until 20 mL (19 mL). The extract was vortexed for 1 min and shaken in AGYTAX® for 5 min at 35°C. Then, the sample was centrifugated (3700 rpm) for 5 min. As final step, extracts were transferred into a 15 mL Falcon tube.

#### Beebread and beeswax

Sample treatment was based on the Quick Polar Pesticide Method for products of Plant Origin (QuPPe-PO-Method) [35], with some modifications. Two grams of sample was weighed into a 50 mL PTFE centrifuge tube and after 10 mL of water was added. The following step was vortexed the sample for 1 min and shaken for 10 min in AGYTAX®. Then, the extracts were centrifuged (4000 rpm) for 30 min. The extract was transferred into a 15 Falcon tube containing 100 mg of Bondesil-C_18_ and 2 mL of Acetonitrile and was then vortexed for 1 min. Extracts were centrifuged (3700 rpm) for 5 min. Finally, the supernatant was transferred into a 15 Falcon tube.

#### Vial preparation

Different procedures were followed according to the matrix. For beebread and beeswax, samples were diluted with ultrapure water to a final dilution factor of 100. In the case of honey and adult bees and bee brood, samples were diluted to a final dilution factor of 50 prior to injection. 2,4-D^13^C6 was used as injection internal standard in all vials for IC-HRMS analysis.

### Validation study

The developed methods for the analysis of FA and OA in beekeeping matrices were validated. The diluted and least contaminated extracts from the apicultural matrices were used for the validation. Validation study was performed regarding sensitivity (method limit of quantification and instrumental limit of detection), recovery (%), linearity, sensitivity, matrix effect (ME), and precision. The limit of quantification (LOQ) was established as the lowest limit of the calibration curve that complied with the criteria to be identified [36]. Instrumental detection limits (IDL) were determined by direct injection of decreasing amounts of the standards and were defined as the minimum detectable amount of analyte with a signal-to-noise ratio of 3:1. The linearity was evaluated by calculating the variation coefficient (*R*^2^), which should be higher than 0.99 and residuals were below 20%. The calibration curves were established using the diluted matrix extracts as well as in milliQ water by injecting 6 concentration levels of FA ranging from 5 to 200 mg/kg in bee matrices and from 20 to 1000 mg/kg in the other beekeeping matrices. In the case of OA ranging from 20 to 1000 mg/kg in all the matrices. Recoveries were calculated for each matrix spiked with the analytes at a concentration of 50 mg/kg. To determine extraction recoveries, concentrations of the spiked matrices before and after extraction were compared. The precision of the methods is represented as the relative standard deviation (% RSD) from the extraction replicates (*n* = 5). Matrix effect was calculated as the percentage decrease or increase in signal intensity in sample matrix versus pure milliQ water. Matrix effect was calculated using the following equation:

$$ME\left(\%\right)=\left(\frac{slope\;of\;calibration\;curve\;standard\;in\;matrix}{slope\;of\;calibration\;curve\;standard\;in\;milli\;Q\;water}-1\right)x100$$

### Analysis by IC-HRMS

For the IC separation, Thermo Scientific^TM^ Dionex^TM^ Integrion^TM^ HPIC^TM^ (Thermo Fisher Scientific, San Jose, USA) system was used. Water and KOH were used as mobile phase. The concentration of KOH was increased for creating the gradient that started at 5 mM KOH and increased to 20 mM in 8 min; from 8 to 12 min, KOH increased to 60 mM and it was maintained until 22 min. At 22.1 min, KOH decreased to 5 mM and constant over 4 min for re-equilibration. Separation was carried out on AS19 and AS11-HC columns for formic and oxalic acids, respectively. In the case of AS19 column, the length, diameter, and particle size were 250 mm, 2 mm, and 4 µm, respectively. A guard column was used to protect the column (Dionex IonPac AG19). The column was thermostatted at 40°C. The length, diameter, and particle size of the AS11-HC column were 250 mm, 2 mm, and 4 µm, respectively. The injection volume was 50 µL. The autosampler was thermostatted at 15 °C. The mobile phase flow was 0.35 mL min^−1^. To neutralize KOH and to convert salts into acids, an AERS 500es 2 mm suppressor was set to 52 mA. The regenerating water flow was 0.6 mL min^−1^. Post column organic solvent (acetonitrile) flow rate was 0.2 mL min^−1^.

The IC was coupled to a Thermo Scientific™ Orbitrap Exploris™ 240 (Thermo Fisher Scientific, Bremen, Germany) mass spectrometer equipped with a Thermo Scientific™ OptaMaxTM NG (H-ESI II) ion source. The ion source parameters in negative polarity were as follows: spray voltage, static; negative ion (V), 1000 and 2500 for FA and OA, respectively; sheath gas flow rate, 40 (arbitrary units); auxiliary gas flow rate, 10 (arbitrary units); sweep gas flow rate, 0 (arbitrary units); ion transfer tube temperature, 280 °C; vaporizer temperature, 300 °C. In this study, two acquisition methods (workflows) were used for OA analysis. Nevertheless, FA only was acquired in SIM mode.

Method A, for FA analysis, included one experiment in MS (Full Scan MS and Selected Ion Monitoring, SIM) mode. Targeted SIM scan properties were as follows: isolation window, m/z 2; orbitrap resolution, 240000 (at m/z 200); RF lens, 70%; the Automatic Gain Control (AGC) target was set to standard; maximum Injection Time (max IT), auto; Microscans, 1.

Method B, for OA, both MS and targeted MS^2^ (tMS^2^) modes were used simultaneously. Targeted SIM scan properties were the same as in Method A. Acquisition parameters in tMS^2^ for OA were as follows: multiplex ions, inactive; isolation window, m/z 2; isolation offset, off; collision energy mode, fixed; collision energy type, absolute; orbitrap resolution, 15,000; scan range, auto; AGC target, standard; maximum Injection Time, auto; microscans, 1. A collision energy of 12 V was selected for oxalic acid. The following ion was used for detection and identification of formic acid: m/z 44.9982 that was obtained in SIM mode. However, oxalic acid was detected in SIM mode (m/z 88.988) and identified in tMS2 (m/z 60.9931).

RunStart EASY-IC was used for mass accuracy calibration. Besides the EASY-IC, the external Mass Calibration with FlexMix solution was carried out once a week and System Calibration once a month. Thermo Scientific^TM^ TraceFinder 5.1 Software (Thermo Fisher Scientific, San Jose, USA) was used for qualitative and quantitative analysis. Automatic detection and quantification were followed up by manual verification. The extraction window and mass tolerance were 5 ppm.

## Results and discussion

### IC-HRMS analysis

The highly polar analytes OA and FA present a challenge for analysis. IC is particularly suitable for the analysis of small organic anions and cations. Here, we present a new approach for the analysis of these compounds in beekeeping matrices, based on ion chromatography coupled to high-resolution mass spectrometry. This methodology is fast and simple and an advantage of HRMS is the ability to measure exact masses of detected ions with errors < 2 ppm and high mass resolution, which are important parameters to avoid false negative and false positive results and to improve the quality of the quantitation, this is especially important for the complex beekeeping matrices of the present study. This method is a significant improvement over previously published methods, where non-selective detectors or derivatization are used. In addition, there are very few methods for the determination of OA and FA in beehive matrices; we have found only references for the analysis of honey or bees [21, 25, 32].

**Fig. 1:**
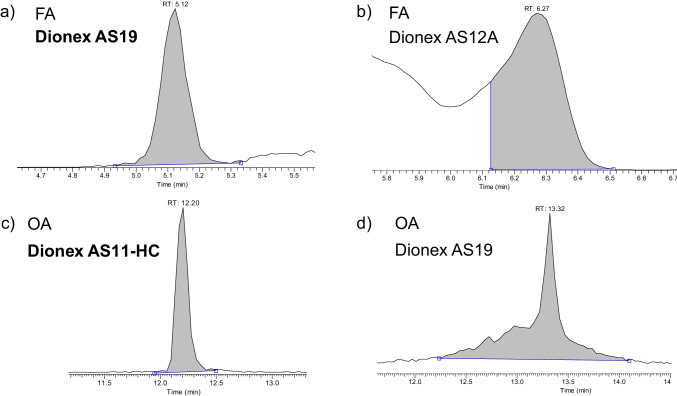
Chromatograms showing the peak shape of FA and OA in beebread with different columns: **a** FA with Dionex AS19; **b** FA with Dionex AS12A; **c** OA with Dionex AS11-HC; **d** OA with Dionex AS19

Formic acid is the most challenging analyte. The difficulties are present in the chromatographic separation as well as in the MS detection. Three anion-exchange columns (Dionex AS11-HC, Dionex AS12A, and Dionex AS19) were tested to find the optimal retention and separation from the matrix. The extracts used for the optimization were spiked with 1 ppm of formic acid and oxalic acid. Figure 1 presents an example of peak shape of FA and OA in beebread with two different columns. With the AS11-HC, no FA peak was observed. The two remaining columns retained FA and provided a good peak shape. Nevertheless, the AS19 column assured a better separation from the different beekeeping matrices, as it can be observed in Fig. 1a, and therefore, it was selected for the validation and sample analysis. The same set of columns was checked with OA. Good retention was obtained with the AS19 and AS11-HC. However, the latter provided a more symmetrical peak and separation from the interferences (Fig. 1c), and consequently, it was chosen for the validation study.

FA is also difficult to analyse by mass spectrometry because it is a very small molecule. By this reason, it is practically impossible to obtain more than one ion. Formate ion (m/z 44.99820) was acquired in SIM MS mode. This acquisition mode provides higher sensitivity than full scan MS [37]. Formic acid did not provide any sensitive fragment in MS^2^ mode. The ^13^C isotope was not helpful because of its low abundance (1.1%). Another problem was related with the selectivity. Formate has a very low m/z; thus, some interferences were present. They were manifested as the elevated baseline. The extremely high resolution (approximately 500,000 at m/z 45) did not reveal any other ions; thus, the interferences had the same formula as formate. It was assumed that the interferences came from the fragmentation of matrix compounds in the ion source. To prevent that fragmentation, the spray voltage was decreased to 1,000V. Oxalic acid was considerably less problematic than formic acid. It formed a single-charged ion detected in SIM mode. That ion was used for the detection. For identification, an MS^2^ fragment ion was employed.

**Fig. 2:**
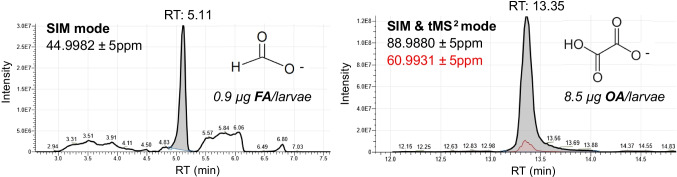
IC-Q-Orbitrap extracted ion chromatograms of FA (left) and OA (right) in larvae real samples

The list of ions and retention times of FA and OA can be found in Table 1. Formic acid CH_2_O_2_ (exact mass: 44.9982) and oxalic acid C_2_H_2_O_4_ (exact mass: 88.9880) with retention time 5.11 and 13.35 min, respectively. In the case of OA, it was confirmed with the fragment ion 60.9931 m/z. The ion ratio from sample extracts was very stable, below 30% in all the cases. An example of the detection of FA and OA by the developed methods in real larvae samples is shown in Fig. 2.

**Table 1:** Retention time (Rt), exact masses, molecular formula, and collision energy of FA and OA ions

	**Rt (min.)**	**MS**		**MS**^**2**^	
Compound		Formula	*m/z*	CE (eV)	*m/z*
Formic acid (FA)	13.35	CH_2_O_2_	44.9982		
Oxalic acid (OA)	5.11	C_2_H_2_O_4_	88.988	12	60.9931

### Sample preparation and methods performance

**Fig. 3:**
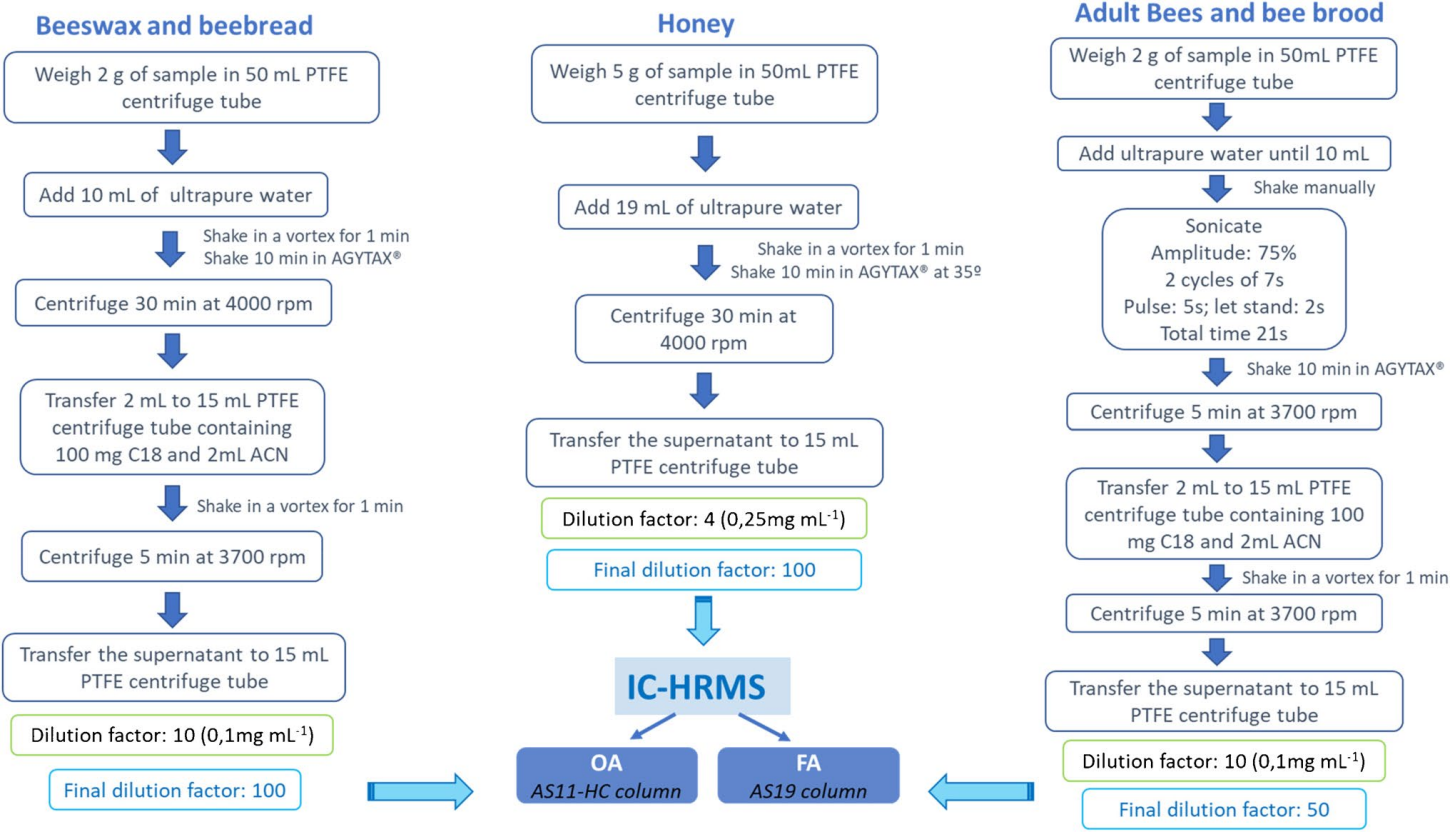
Diagrams of the extraction protocols for FA and OA in the different beekeeping matrices

Different approaches seeking the simplification for the sample preparation step were developed and validated. Figure 3 shows diagrams of the optimized methods for the extraction of FA and OA in the different beekeeping matrices. For sample preparation of all the beekeeping matrices, except for honey samples, QuPPe methods modified with d-SPE (using C_18_) and dilution with ACN were used for the removal of lipids and protein precipitation, respectively. The extraction method of honey samples was a simple dilution with ultrapure water and agitation at 35°C. A modified QuPPe-AO-Method for products of animal origin [33] was not only used for adult bees and bee brood sample preparation, but also was employed an ultrasonic-assisted probe extraction method based on the method proposed by Gil et al. to facilitate the extraction of analytes from the honeybees [34]. The adjustment of water content for these matrices of animal origin has been done considering their natural water content. In order to reach a total water content of 10 g per portion, different volumes of water were added: 8.7 mL for bees, 8.6 mL for larvae, and 8.4 mL for prepupae. A modified QuPPe-PO-Method for food of plant origin [35] was used for beeswax and beebread samples. In both modified QuPPe methods, only water was added without methanol or formic acid. Taking into account the high concentrations of FA and OA in these matrices, mainly due to their natural content, and to minimize matrix effects and interferences, the final extracts were further diluted to a final dilution factor of 50 for bee samples and 100 for honey, beeswax, and beebread samples, prior to injection.

The developed methods were validated mainly following the DG-SANTE guidelines [36]. A brief overview of validation results of the optimized methods is presented in Table 2. Instrumental detection limit (IDL) for FA was 0.1 mg/kg and 0.001 mg/kg for OA. The LOQ value for FA and OA was 20 mg/kg in the studied beehive matrices, except for FA in bee matrices that was 5 mg/kg, which guarantees a correct evaluation of the organic acids in the beehive matrices. Precision and accuracy were evaluated as recoveries and RSD in accordance with DG-SANTE guidelines [36]. All matrices spiked with FA and OA presented recoveries in the acceptable range (70–120%) with an associated precision of RSD < 20 %. Nevertheless, FA in beeswax and beebread matrices showed recoveries lower than 70%. Despite these low recoveries (52% and 47%), the other validation data, such as precision and sensitivity, are good, and therefore, a reliable determination of FA at the concentration levels present in the matrices is feasible. Good linearity was found for both compounds in all matrices in the concentration range considered in this study (*R*^2^ > 0.99). To determine the matrix effect (ME), both calibration curves (in pure water and in matrix-matched) were compared. For FA, signal suppression (ME < −30%) was found in bee and beebread matrices, and in honey (27%) and beeswax (5%), enhancement of the signal was found. For OA, low ME were observed; signal suppression was found in beeswax and the rest of the matrices showed a signal enhancement (ME ≤ 10%). The validation study has proved the suitability of the methods to analyse FA and OA in beekeeping matrices.

**Table 2:** Brief overview of validation results for the analysis of FA and OA in the different beehive matrices

Organic Acaricide	Matrix	*R*^2^	Matrix effect (%)	Recovery, RSD (%)
Formic acid	Bee	0.9993	−28	73 (3)
	Honey	0.9981	27	77 (4)
	Beeswax	1.0000	5	52 (2)
	Beebread	0.9890	−24	47 (2)
Oxalic acid	Bee	0.9998	9	82 (7)
	Honey	1.0000	6	68(8)
	Beeswax	1.0000	−21	67 (16)
	Beebread	0.9992	10	77 (2)

### Field study

Given the difficulty of analysing these organic acids and the lack of robust and reliable methods in the complex beehive matrices, there is very few research on the levels of concentration, distribution, and persistence of these widely used natural acaricides in the beehive compartments during and after treatment application by the beekeeper. To the best of our knowledge, this is the first research in field conditions that evaluates comprehensively the residue levels of these organic acids in the honey-wax-pollen-bee-bee brood system in practical beekeeping.

**Fig. 4:**
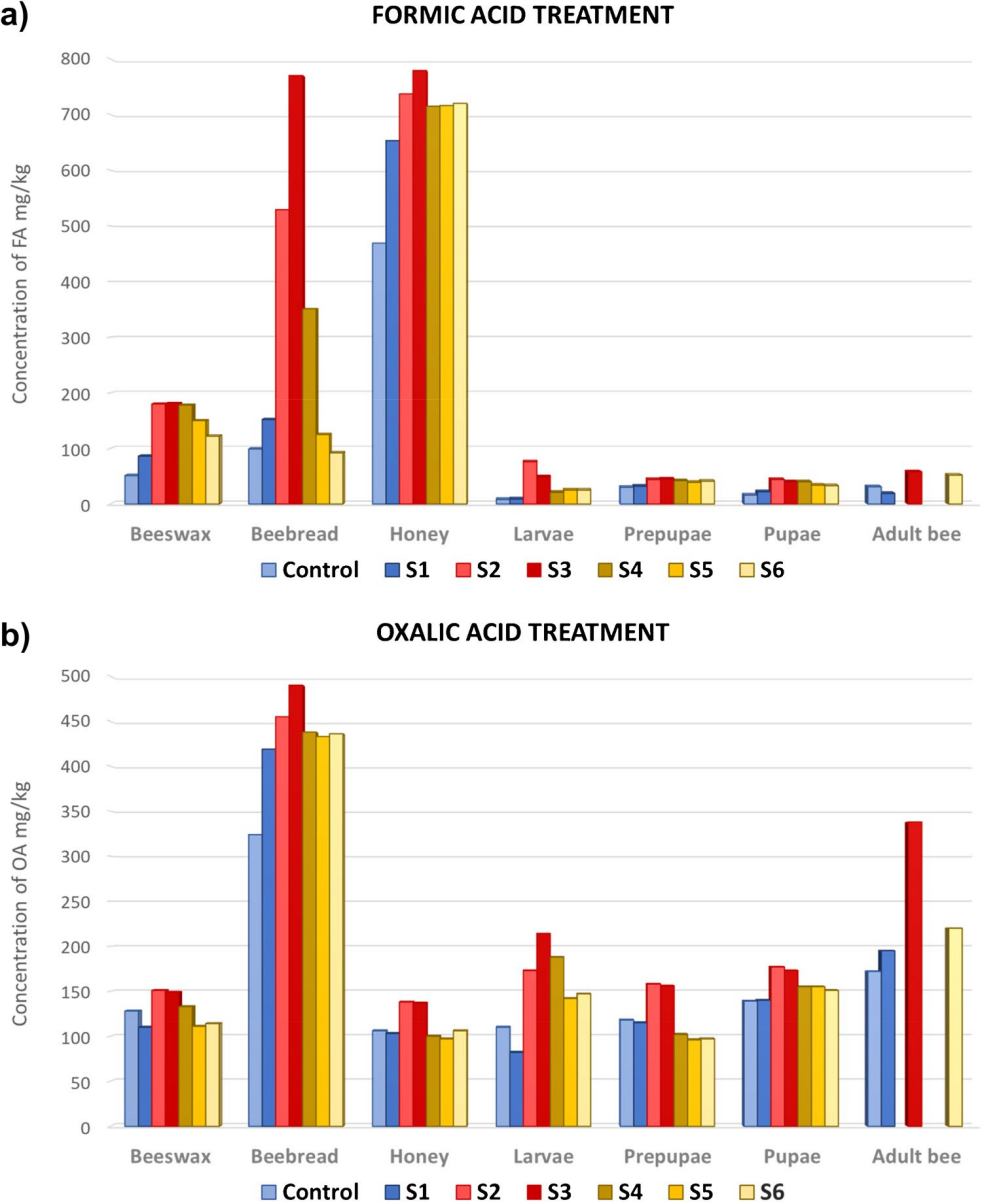
Concentration of **a** FA and **b** OA in bees and the beehive compartments during the field study: Before the treatments (S1), during the treatment (S2, S3), and 1 month (S4), 2 months (S5), and 3 months (S6) after the treatment

The concentrations of FA and OA found in each sampling month for all the studied matrices are presented in Table 3. These results correspond to the average concentration of samples from four honeybee colonies, which were pooled for their analysis. Four honeybee colonies not treated with the organic acids were used as control throughout the study. In Fig. 4, the results of the field study are shown in a graphic. It can be observed the distribution and concentration of FA and OA in the beehive compartments during the study: before, during, and after the treatments.

**Table 3:** Formic acid and oxalic acid residue concentrations found in beehive matrices during the field study

Matrix	Control	Before treatment	During treatment		After treatment			LD_50_
		S1	S2	S3	S4	S5	S6	
Formic acid concentration (mg/kg)								
Beeswax	51	86	180	181	178	150	122	
Beebread	99	152	529	770	351	125	92	
Honey	469	653	737	779	715	716	720	
Larvae *(μg/larvae)*	8 *(0.8)*	9 *(0.9)*	76 *(7.9)*	49 *(5.1)*	21 *(2.2)*	25 *(2.6)*	25 *(2.6)*	
Prepupae *(μg/prepupae)*	30 *(3.8)*	32 *(4.0)*	44 *(5.5)*	45 *(5.7)*	42 *(5.3)*	39 *(4.9)*	41 *(5.2)*	
Pupae *(μg/pupae)*	16 *(1.9)*	22 *(2.6)*	44 *(5.2)*	40 *(4.7)*	40 *(4.7)*	34 *(4.0)*	33 *(3.9)*	
Adult bee *(μg/bee)*	31 *(3.5)*	19 *(2.2)*		58 *(6.6)*			52 *(5.9)*	*(152)*
Oxalic acid concentration (mg/kg)								
Beeswax	128	110	151	149	133	111	114	
Beebread	324	419	455	490	438	433	436	
Honey	106	103	138	137	100	97	106	
Larvae *(μg/larvae)*	110 *(11.4)*	82 *(8.5)*	173 *(17.9)*	214 *(22.3)*	188 *(19.6)*	142 *(14.8)*	147 *(15.3)*	*(45)*
Prepupae *(μg/prepupae)*	118 *(14.9)*	115 *(14.5)*	158 *(19.9)*	156 *(19.7)*	102 *(12.9)*	96 *(12.1)*	97 *(12.2)*	
Pupae *(μg/pupae)*	139 *(16.4)*	140 *(16.5)*	177 *(20.9)*	173 *(20.4)*	155 *(18.3)*	155 *(18.3)*	151 *(17.8)*	
Adult bee *(μg/bee)*	172 *(19.4)*	195 *(22.0)*		338 *(38.2)*			220 *(24.9)*	*(265)*

It can be observed that in all the control samples and samples collected before treatments there is a certain concentration of OA and FA residues, in some matrices quite high. This is probably due to its natural presence in plants, and therefore in beebread and honey.

Concentration levels of FA in beeswax from controls and samples taken before the treatment were 51 mg/kg and 86 mg/kg, respectively. The concentration increased to 181 mg/kg during treatment. After the treatment, the concentration progressively decreases, until reaching a concentration of 122 mg/kg 3 months after treatment. This means a percentage of disappearance of more than 60% of the FA residues added by the treatment, in 3 months. In the case of OA, concentrations of 128 mg/kg and 110 mg/kg were observed in the control samples and in the first sampling, before the treatment administration, respectively. Although there was a small increase in OA residues during the treatment administration, 2 months later, the levels of OA in the beeswax diminished to reach the levels before treatment application. Residue levels of these natural acaricides in the control samples and in the samples taken before the treatment could indicate that an amount of these organic acids could persist in the wax for some time. However, as organic acids are hydrophilic substances, they have low persistence in wax, and it is more probable that waxes have constant exposure to FA and OA through pollen, honey, and other bee matrices where OA and FA are naturally present (see the levels of FA and OA residues in beebread and honey in Fig. 4).

Honey is the highest reservoir of FA. As it is shown in Table 3 and Fig. 4, the honeys from the untreated colonies (controls and S1) had a high FA content (469–653 mg/kg). Figure 4 shows a moderate increase in concentration during the application of the treatment and a slow dissipation that remains practically constant after application (715–720 mg/kg). This increment of FA is not very important as it is very close to the natural concentration of this acid in honey. Natural levels of FA have been found in honeys in the range of 9 to 1229 mg/kg [21, 23]. Previous studies, carried out under controlled experimental conditions after treatments with FA, also have found that the residue levels in honey were within the natural variation of this acid [23, 24]. The concentration levels of the OA in honey were considerably lower than those of the FA, and they remained quite stable throughout the study, in the range of 103 to 138 mg/kg. This is consistent with other studies, where no significant increase of oxalic acid concentration in honey was observed during and after oxalic acid treatments [23]. According to the EU residue regulation, OA and FA are considered safe, and the establishment of MRL is not required to protect food safety [19].

Measuring residues in beebread is crucial as is considered one of the main sources of exposure for honeybees. Beebread is a mixture of pollen, propolis, honey, and other bee substances that are able to retain lipophilic and hydrophilic substances. As can be observed in Fig. 4 and Table 3, beebread is the matrix with the higher content in OA. The concentration of OA in beebread is 324 mg/kg in the control and 419 mg/kg in the samples taken before the treatment, indicating that OA is a compound that occurs naturally in this matrix. This concentration increases in the samples taken during the treatment, with S3 having a concentration of 490 mg/kg. Dissipation after treatment remains practically constant during the three months after OA administration. However, taking into account the relatively high levels of residues in the untreated samples, the increase due to the treatment is not significant, indicating that this matrix is not greatly affected by the treatment with oxalic acid. In the case of FA, residues in beebread from the control and untreated hives were much smaller than those found in honey before treatment. Nevertheless, FA concentration increased considerably during the treatment application up to 770 mg/kg, but after removal of treatment strips, the levels decreased rapidly, until reaching the values observed before treatment.

Adult bee and bee brood are the beekeeping matrices with the lowest concentration of FA (Fig. 4). During the treatment application, they experimented a slight residue increase, more significant in the case of larvae (from 9 to 76 mg/kg). A slow disappearance of FA was observed after the treatment. Compared with FA, OA was found at higher concentrations in adult bees and bee brood. Adult bees presented the highest increment of residues during the treatment administration, but 3 months after application, concentration of OA decreased to levels close to those found before treatment. It can be assumed that this increase during the treatment application could be produced by the way in which this treatment is administered, by pouring it with a syringe directly to the bees. As in the case of FA, residues of OA in prepupae and pupae were quite stable during the study, with a slight increase in concentration values during treatment (S2 and S3). A more significant increase in concentration was also observed in the larvae during treatment.

According to the results obtained, only a small portion of FA is transferred to bee brood or adult bees. OA is presented at higher concentrations in adult and developing bees; however, the increase in OA produced by the treatment does not seem very significant, considering the concentrations before the treatment and from untreated beehives. This may be due to the bees’ ingestion of beebread, which contains high concentration of OA (Fig. 4).

Gashout et al. evaluated the toxicity of organic and synthetic acaricides to adult honeybees [8]. They showed that natural acaricides were significantly less toxic to bees than the synthetic ones, with FA having the lowest LD50 value (152 ug/bee) [8]. This value is about 20 times higher than the maximum concentration found in our study for adult and developing bees. The toxicity of OA to larvae and adult bees was determined by Sabová et al. [38]. OA had the lowest LD50 (72 h) for larvae, with a value of 45 μg OA/larva. In adult bees, the calculated LD50 was 265 μg OA/bee. In this study, the maximum concentration of OA found in adult bees and larvae were 38 and 22 μg per body, respectively (Table 3). In the case of adult bees, values are about 10 times lower than the reported LD50. However, OA concentration in larvae samples during the treatment application is very closed to the reported LD50 value. These data indicate that the organic acids residues during and after the application of the treatments were in all cases below the reported toxicity levels in adult and immature bees; however, the possibility that the application of the OA could pose a risk to larval development cannot be ignored.

## Conclusions

An effective and robust approach for the analysis of the highly polar organic acaricides in bees and beehive products has been developed. It is based on sample extraction with modified QuPPe methods followed by IC-HRMS analysis. The developed methods were validated, proving their suitability to analyse FA and OA in the beekeeping matrices. The methodology showed to be fast and selective, and the measurement of accurate mass provides a high level of confidence for the determination of OA and FA in the beehive matrices, being an important improvement to the previously used methods.

In the field study, residues of FA and OA have been found in all the control samples and samples collected before the treatments, being quite high in honey (FA) and beebread (OA), mainly due to its natural presence in plants. These matrices showed an increase of organic acid concentration during the treatment but, taking into account the levels of residues before the treatment, the increase due to the treatment was not significative. The organic acids showed low persistence in wax after the treatments; however, they have constant exposure to FA and OA, most probably through beebread and honey contact, where they are naturally present. Only a small portion of FA (8–76 mg/kg) is transferred to bee brood or adult bees. OA is presented at higher concentrations in adult and developing bees (82–338 mg/kg); however, the increase in OA produced by the treatment does not seem very significant, considering the concentrations before the treatment and from untreated beehives. With both treatments, larvae were the bee brood that experiment the most significant residue increment during treatment administration. FA and OA residues during and after the application of the treatments were in all cases below the reported toxicity levels in adult bees and bee brood. In the case of FA, residues were about 20 times lower than the LD50 value, but OA concentration levels were closer to the calculated LD50, especially in larvae samples during the treatment application.

## References

[1] Guichard M, Dietemann V, Neuditschko M, Dainat B (2020). Advances and perspectives in selecting resistance traits against the parasitic mite Varroa destructor in honey bees. Genet Sel Evol.

[2] Hung KLJ, Kingston JM, Albrecht M, Holway DA, Kohn JR (2018) The worldwide importance of honey bees as pollinators in natural habitats. Proceedings of the Royal Society B: Biological Sciences 285: . 10.1098/rspb.2017.2140 10.1098/rspb.2017.2140 PMC5784195 29321298

[3] Tihelka E (2018). Effects of synthetic and organic acaricides on honey bee health: a review. Slovenian Veterinary Research.

[4] Neumann P, Carreck NL (2010). Honey bee colony losses. J Apic Res.

[5] Haber AI, Steinhauer NA, Vanengelsdorp D (2019). Use of chemical and nonchemical methods for the control of Varroa destructor (Acari: Varroidae) and associated winter colony losses in U.S. beekeeping operations. J Econ Entomol.

[6] Rosenkranz P, Aumeier P, Ziegelmann B (2010). Biology and control of Varroa destructor. J Invertebr Pathol.

[7] Gunes N, Aydın L, Belenli D, Hranitz JM, Mengilig S, Selova S (2017). Stress responses of honey bees to organic acid and essential oil treatments against varroa mites. J Apic Res.

[8] Gashout HA, Goodwin PH, Guzman-Novoa E (2018). Lethality of synthetic and natural acaricides to worker honey bees (Apis mellifera) and their impact on the expression of health and detoxification-related genes. Environ Sci Pollut Res.

[9] Boi M, Serra G, Colombo R, Lodesani M, Massi S, Costa C (2016). A 10 year survey of acaricide residues in beeswax analysed in Italy. Pest Manag Sci.

[10] Dai P, Jack CJ, Mortensen AN, Ellis JD (2017). Acute toxicity of five pesticides to Apis mellifera larvae reared in vitro. Pest Manag Sci.

[11] Murcia Morales M, Gómez Ramos MJ, Parrilla Vázquez P, Díaz Galiano FJ, García Valverde M, Gámiz López V, Manuel Flores J, Fernández-Alba AR (2020). Distribution of chemical residues in the beehive compartments and their transfer to the honeybee brood. Sci Total Environ.

[12] Bogdanov S, Charrière JD, Imdorf A, Kilchenmann V, Fluri P (2002). Determination of residues in honey after treatments with formic and oxalic acid under field conditions. Apidologie.

[13] Manzano Sánchez L, Gómez Ramos MJ, del Maria GRM, Parrilla Vazquez P, Flores JMR, Fernández-Alba A (2021). Presence, persistence and distribution of thymol in honeybees and beehive compartments by high resolution mass spectrometry. Environmental Advances.

[14] Kasiotis KM, Tzouganaki ZD, Machera K (2018). Chromatographic determination of monoterpenes and other acaricides in honeybees: prevalence and possible synergies. Sci Total Environ.

[15] Nozal MJ, Bernal JL, Jiménez JJ, González MJ, Higes M (2002). Extraction of thymol, eucalyptol, menthol, and camphor residues from honey and beeswax: determination by gas chromatography with flame ionization detection. J Chromatogr A.

[16] Tonello N, Moressi MB, Robledo SN, D’Eramo F, Marioli JM (2016). Square wave voltammetry with multivariate calibration tools for determination of eugenol, carvacrol and thymol in honey. Talanta.

[17] Charpentier G, Vidau C, Ferdy JB, Tabart J, Vetillard A (2014). Lethal and sub-lethal effects of thymol on honeybee (Apis mellifera) larvae reared in vitro. Pest Manag Sci.

[18] Gao H, Cao W, Liang Y, Cheng N, Wang BN, Bin ZJ (2010). Determination of thymol and phenol in honey by LC with electrochemical detection. Chromatographia.

[19] European Commission 2010 (2010) Commission Regulation (EU) No 37/210 on pharmacologically active substances and their classification regarding maximum residue limits in foodstuffs of animal origen. https://eur-lex.europa.eu/legal-content/EN/TXT/PDF/?uri=CELEX:32010R0037&from=EN

[20] Maggi M, Tourn E, Negri P, Szawarski N, Marconi A, Gallez L, Medici S, Ruffinengo S, Brasesco C, De Feudis L, Quintana S, Sammataro D, Eguaras M (2016). A new formulation of oxalic acid for Varroa destructor control applied in Apis mellifera colonies in the presence of brood. Apidologie.

[21] Matysiak I, Balcerzak M, Michalski R (2018). Ion chromatography with conductometric detection for quantitation of formic acid in Polish bee honey. J Food Compos Anal.

[22] Gashout HA, Guzman-Novoa E, Goodwin PH (2020). Synthetic and natural acaricides impair hygienic and foraging behaviors of honey bees. Apidologie.

[23] Abdullah I, Gary SR, Marla S (2007). Field trial of honey bee colonies bred for mechanisms of resistance against Varroa destructor. Apidologie.

[24] Mato I, Huidobro JF, Simal-Lozano J, Sancho MT (2003). Significance of nonaromatic organic acids in honey. J Food Prot.

[25] Kasiotis KM, Manea-Karga E, Machera K (2019) A zwitterionic hydrophilic interaction liquid chromatographic photo diode array method as a tool to investigate oxalic acid in bees: comparison with mass spectrometric methods. Separations 6: . 10.3390/separations6040048

[26] Showler AT, Dorsey BN, Caesar RM (2020). Effects of formic acid on amblyomma americanum (Ixodida: Ixodidae) larvae and nymphs. J Med Entomol.

[27] Gregorc A, Alburaki M, Sampson B, Knight PR, Adamczyk J (2018) Toxicity of selected acaricides to honey bees (Apis mellifera) and varroa (varroa destructor anderson and trueman) and their use in controlling varroa within honey bee colonies. Insects 9: . 10.3390/insects9020055 10.3390/insects9020055 PMC6023343 29748510

[28] Fang Y, Xu X, Guo X, Cui B, Wang L (2020). Simple and ultrasensitive electrochemical sensor for oxalic acid detection in real samples by one step co-electrodeposition strategy. Anal Bioanal Chem.

[29] Balcerzak M, Kapica D (2017). Fast ion chromatographic method for the determination of formates in alcoholic drinks. Food Anal Methods.

[30] Rajski Ł, Díaz Galiano FJ, Cutillas V, Fernández-Alba AR (2018). Coupling ion chromatography to Q-orbitrap for the fast and robust analysis of anionic pesticides in fruits and vegetables. J AOAC Int.

[31] Pareja L, Jesús F, Heinzen H, Hernando MD, Rajski Ł, Fernández-Alba AR (2019). Evaluation of glyphosate and AMPA in honey by water extraction followed by ion chromatography mass spectrometry. A pilot monitoring study. Analytical Methods.

[32] Nozal MJ, Bernal JL, Gómez LA, Higes M, Meana A (2003). Determination of oxalic acid and other organic acids in honey and in some anatomic structures of bees. Apidologie.

[33] Anastassiades M, Wachtler A-K, Kolberg DI, Eichhorn E, Benkenstein A, Zechmann S, Mack D, Barth D (2019). EU Reference Laboratory for pesticides requiring Single Residue Methods (EURL-SRM) quick method for the analysis of numerous highly polar pesticides in food involving extraction with acidified methanol and LC-MS/MS measurement II. Food of Animal Origin (Q. Eurl-Srm.

[34] Gil García MD, Martínez Galera M, Uclés S, Lozano A, Fernández-Alba AR (2018). Ultrasound-assisted extraction based on QuEChERS of pesticide residues in honeybees and determination by LC-MS/MS and GC-MS/MS. Anal Bioanal Chem.

[35] Anastassiades M, Kolberg DI, Eichhorn E, Wachtler A-K, Benkenstein A, Zechmann S, Mack D, Wildgrube C, Barth A, Sigalov I, Görlich S, Dörk D, Cerchia G (2020). Quick method for the analysis of numerous highly polar pesticides in food involving extraction with acidified methanol and LC-MS/MS measurement. Eurl-Srm.

[36] SANTE/12682/2019 (2019) Guidance document on analytical quality control and method validation for pesticide residues analysis in food and feed. Safety of the Food Chain Pesticides and Biocides European Commission 1–48

[37] Rajski Ł, Martínez-Bueno MJ, Ferrer C, Fernández-Alba AR (2019). LC-ESI-QOrbitrap^TM^ MS/MS within pesticide residue analysis in fruits and vegetables. TrAC - Trends in Analytical Chemistry.

[38] Sabová L, Sobeková A, Staroň M, Sabo R, Legáth J, Staroňová D, Lohajová Ľ, Javorský P (2019). Toxicity of oxalic acid and impact on some antioxidant enzymes on in vitro–reared honeybee larvae. Environ Sci Pollut Res.

